# Comparative Study of γ- and e-Radiation-Induced Effects on FBGs Using Different Femtosecond Laser Inscription Methods

**DOI:** 10.3390/s21248379

**Published:** 2021-12-15

**Authors:** Antreas Theodosiou, Arnaldo Leal-Junior, Carlos Marques, Anselmo Frizera, Antonio J. S. Fernandes, Andrei Stancalie, Andreas Ioannou, Daniel Ighigeanu, Razvan Mihalcea, Constantin Daniel Negut, Kyriacos Kalli

**Affiliations:** 1 Photonics and Optical Sensors Research Laboratory (PhOSLab), Cyprus University of Technology, Saripolou 33, Limassol 3036, Cyprus; andrg.ioannou@edu.cut.ac.cy (A.I.); kyriacos.kalli@cut.ac.cy (K.K.); 2 Graduate Program in Electrical Engineering, Federal University of Espirito Santo, Fernando Ferrari Avenue, Vitoria 29075-910, Brazil; leal-junior.arnaldo@ieee.org (A.L.-J.); frizera@ieee.org (A.F.); 3 i3N, Department of Physics, University of Aveiro, Campus Universitário de Santiago, 3810-193 Aveiro, Portugal; carlos.marques@ua.pt (C.M.); toze2@ua.pt (A.J.S.F.); 4 Center for Advanced Laser Technologies, National Institute for Laser, Plasma and Radiation Physics, 409 Atomistilor St., RO-077125 Magurele, Romania; andrei.stancalie@inflpr.ro (A.S.); daniel.ighigeanu@inflpr.ro (D.I.); razvan.mihalcea@inflpr.ro (R.M.); 5 “Horia Hulubei” National Institute for R&D in Physics and Nuclear Engineering, 30 Reactorului St., RO-077125 Magurele, Romania; dnegut@nipne.ro

**Keywords:** radiation hardness, FBGs, femtosecond laser inscription, gamma radiation, electron radiation, optical sensors

## Abstract

This work presents an extensive, comparative study of the gamma and electron radiation effects on the behaviour of femtosecond laser-inscribed fibre Bragg gratings (FBGs) using the point-by-point and plane-by-plane inscription methods. The FBGs were inscribed in standard telecommunication single mode silica fibre (SMF28) and exposed to a total accumulated radiation dose of 15 kGy for both gamma and electron radiation. The gratings’ spectra were measured and analysed before and after the exposure to radiation, with complementary material characterisation using Fourier transform infrared (FTIR) spectroscopy. Changes in the response of the FBGs’ temperature coefficients were analysed on exposure to the different types of radiation, and we consider which of the two inscription methods result in gratings that are more robust in such harsh environments. Moreover, we used the FTIR spectroscopy to locate which chemical bonds are responsible for the changes on temperature coefficients and which are related with the optical characteristics of the FBGs.

## 1. Introduction

Fibre Bragg gratings (FBGs) are widely used in many applications as sensing elements [1,2]. They are often preferred to electrical sensors due their small size and flexible design, offering immunity to electromagnetic interference and their unique multiplexing capabilities. FBGs are wavelength selective filters that reflect light from the laser-induced periodic refractive index modulations in the core of the fibre that satisfy the Bragg condition at a specific resonant wavelength, $\lambda_{Bragg},$ as shown below,

(1) $$\lambda_{Bragg}=2n_{eff}\Lambda \;\;\;$$

where $n_{eff}$ is the effective refractive index and Λ is the modulation period. These optical components are intended to be used either as radiation or temperature sensors in harsh radiation-ionising environments. The temperature response of the FBGs is denoted as

(2) $$\lambda_{B}\left(T\right)=\lambda_{B}\left(T_{0}\right)+\alpha_{0}\left(T-T_{0}\right)\;$$

where $\alpha_{0}$ is the FBG temperature sensitivity at a temperature $T_{0}$, which typically is ≈10 pm/°C for gratings inscribed in germanosilicate fibres. For radiation sensing measurements, the most studied mechanism is the radiation-induced attenuation (RIA), which is related with the absorption bands of the material [3]. RIA is influenced by the amount of germanium (Ge) doping in the fibre core [4,5]. Increasing the dopant in the fibre core is a common technique for increasing the fibre photosensitivity, making the fibre more sensitive to ultraviolet (UV) laser modification of the refractive index for the inscription of the FBGs. An alternative is to combine exposure to UV laser light with hydrogenation to modify the optical fibre core, resulting in highly radiation sensitive Type IA FBGs [6,7]. Pure silica fibres have been shown robust performance and limited RIA effects, enabling applications for temperature measurements in radiation environments [8]. However, by minimising or removing the dopants in the silica core implies a non-photosensitive fibre, impeding or preventing FBG inscription, unless deep-UV lasers are used.

An alternative solution to efficient grating inscription is the use of high-intensity ultra-fast pulses derived from femtosecond lasers. The femtosecond laser-inscribed FBGs either using UV, visible, or infrared (IR) radiation, are characterised by having greater temperature stability and measurement capability close to 1000 °C. In addition, as Gusarov et al. [9] showed, when the femtosecond laser inscription is performed via a two-photon absorption process, the gratings under ionised radiation have very similar wavelength shifts, either using pure silica or heavily doped silica fibres [10]. The femtosecond laser inscription locally increases the material density, resulting in greater radiation resistance.

In addition to the RIA effect, another effect can also induce refractive index changes when glass is exposed to high-energy particles or ionising radiation, causing the radiation-induced refractive index change, which is well known as RIRIC [11,12]. One of the physical explanations regarding this effect was due to the compaction phenomenon and appearance of defect-related absorption bands via Kramers–Kronig relations [13].

**Figure 1:**
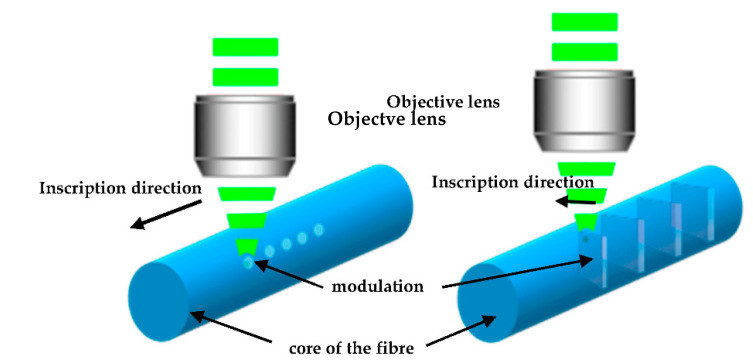
Schematic diagram of the refractive index modification according to the inscription method; (**left**) point-by-point and (**right**) plane-by-plane method.

There are four key femtosecond laser inscription methods. The first method is inscription using a phase mask [14,15], the second is the point-by-point (*PbP*) method [16,17,18], where high-intensity pulses generate periodic points in the core of the fibre, the line-by-line method [19,20] and the plane-by-plane (*PlbPl*) method [21,22,23,24]. The *PbP* method employs tightly focused fs laser pulses to form microscale voids in the centre of the core. The gratings are realised by a series of voids with high-precision positioning in the fibre’s core, with a distance between two consecutive voids equal to the grating period. On the other hand, using the *PlbPl* method, three-dimensional refractive index planes are created with uniform modification distributed across the core with lower pulse energy. This does not affect the optical profile of the FBGs, but it affects the material modification and the interaction of the radiation with the laser-modified material. A schematic diagram showing the induced refractive index modification in the fibre core for the *PbP* and *PlbPl* is presented in Figure 1. 

In 2015, Morata et al. [25] studied the response of type II femtosecond laser-written FBGs using the phase mask and the PbP methods when exposed to X-ray radiation. The *PbP*-FBG have been found to have more stable performance when compared with the phase mask FBGs with respect to Bragg wavelength shift and amplitude variation of the gratings. This work was the first one that studied the radiation hardness of *PbP*-FBGs. The same group confirmed their results in 2017 [26], concluding that the specific gratings showed the best hardness to radiation environments. In this paper, we compare the *PbP* and *PlbPl* methods when the gratings were exposed to γ- and e-radiation, and we study the permanent changes of the FBGs after the exposure. In addition, we proceed a step further and give a first consideration of how these changes occur at a molecular level, influencing the temperature sensitivity of the FBGs, with respect to Fourier Transform infrared (FTIR) spectroscopy results. To the best of our knowledge, this is the first work comparing the post-irradiation effects of femtosecond written gratings on exposure to γ- and e-radiation. These comparative studies are necessary to understand the possibilities of optimising sensors for their possible use in harsh environments. 

## 2. Materials and Methods

### 2.1. Inscription Details

The FBGs used for this work were all inscribed using a femtosecond laser (HighQ) operating at 517 nm with 220 fs laser pulse duration. We used standard Corning SMF28 fibre with an 8.2 μm core diameter, 125 μm cladding diameters, and 3% mol germanium content in the core. We inscribed gratings operating at ≈1560 nm using two different inscription methods, *PlbPl* and *PbP*. It is noted that all the inscriptions were performed through the polymer fibre coating.

**Figure 2:**
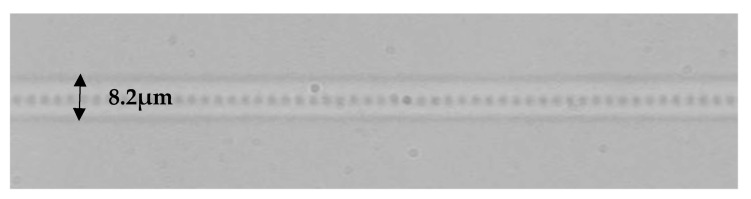
Microscope image of an FBG inscribed using the *PbP* femtosecond laser inscription method.

For the *PbP* inscriptions, the pulse energy was set at ≈900 nJ, and the repetition rate was set at 1 kHz. The fibre samples were mounted on an air-bearing translation stage (Aerotech) with nanometre accuracy for accurate movement during the inscription while the laser pulse focused through a long-working distance objective, with NA 0.42. The translation speed was calculated and set according to the period of the gratings; for a fourth-order FBG with a resonance wavelength at 1560 nm, the equivalent period is ≈2.15 μm. A single pulse train was inscribed at the centre of the fibre core on the fibre axis, with a total grating length of ≈7 mm, as shown in Figure 2. 

**Figure 3:**
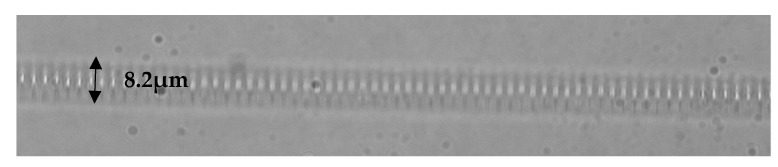
Microscope picture of the refractive index modifications induced using the *PlbPl* femtosecond laser inscription method.

**Figure 4:**
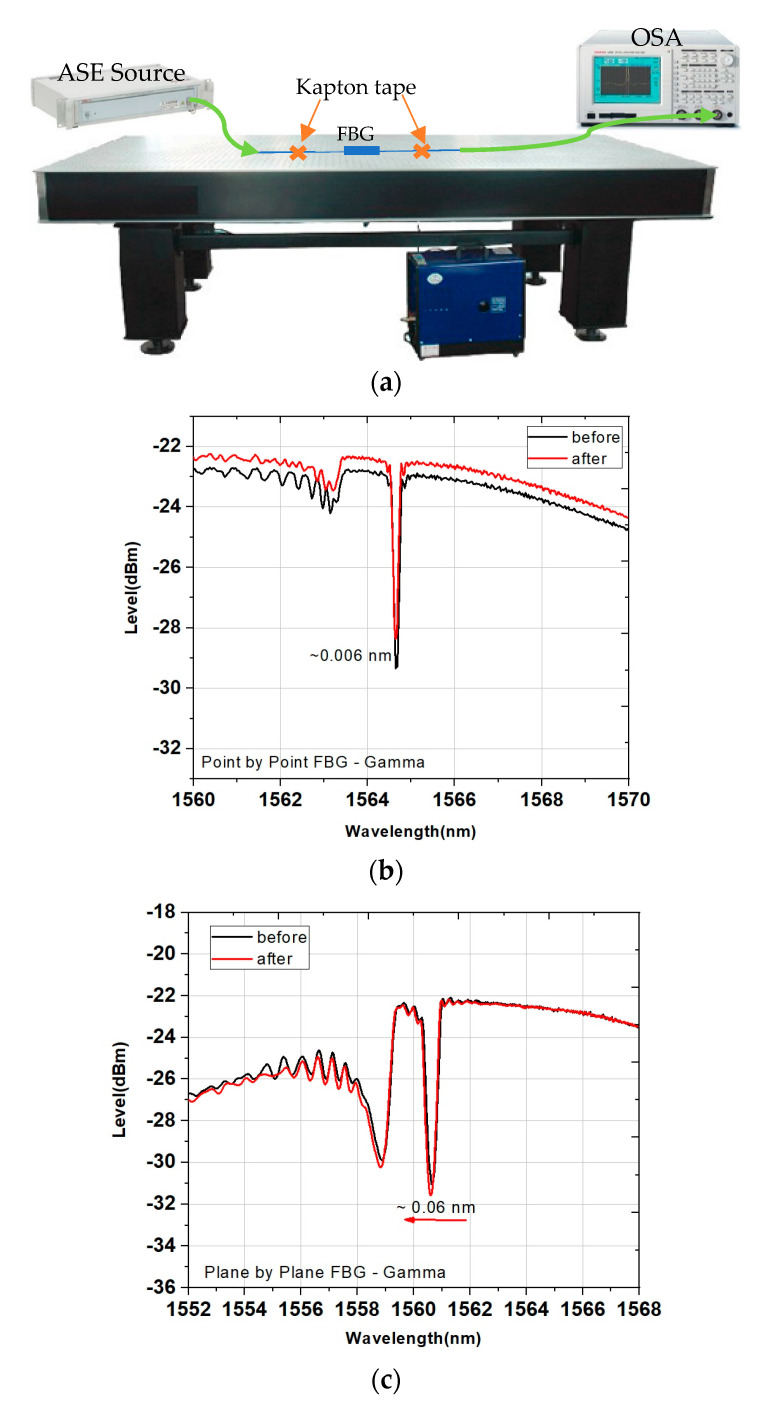
(**a**) Experimental schematic diagram of the FBG transmission measurements. Transmission spectra of the FBG before and after the exposure to γ-radiation (**b**) for the *PbP*-FBG and (**c**) for the *PlbPl*-FBG.

The same inscription setup was used for the *PlbPl* inscription method, using a pulse energy of ≈100 nJ, inscribing refractive index modification planes perpendicular to the fibre core, as presented in Figure 3. The rest of the inscription parameters were as follows: repetition rate of 5 kHz and 1000 grating periods, which is equivalent to ≈2.58 mm total grating length. A fundamental difference between the *PlbPl* and *PbP* method is required for higher energy pulses for the inscription of strong gratings in the latter method. All the FBG samples were spliced with APC/FC silica pigtails and characterised in transmission, prior to subjecting them to any radiation experiments, using a broadband light source, ASE730 (Thorlabs) and optical spectrum analyser (OSA), Advantest Q8384 with 10 pm optical resolution (Figure 4a).

### 2.2. Radiation Exposure Details

The gamma irradiation of the samples was performed using the ^60^Co GC-5000 (BRIT, India) irradiator of the “Horia Hulubei” National Institute of Physics and Nuclear Engineering, having an irradiation chamber volume of 5000 cm^3^. The setup is described in detail in [27]. For the irradiation requirements, all samples were concentrically spooled to a diameter of about 10 cm, separately mounted between two cardboard sheets, and placed in the middle of the cylindrical irradiation chamber, 10 cm from the base of the chamber. The maximum temperature during the exposure was 32 °C, increasing by a mean slope of 0.1 °C/min, starting from room temperature. This did not affect the experiments, as we investigated the results following exposure. The dose rate was 5.5 kGy/h, which was measured with one standard deviation of 3.3%. The total accumulated doses for the four optical fibre samples were 15 kGy. The dosimetry system employed was of the Ethanol-Chlorobenzene (ECB) type with oscillometric readout, traceable at the National Physical Laboratory, by RISOE HDRL.

The electron beam irradiation was performed in several steps, using a “travelling-wave” linear accelerator. The accelerator was driven by a 2 MW peak power tunable EEV M5125 type magnetron operating in the S-band (2992–3001 MHz). The optimum values of the electron beam (EB) peak current I_EB_ and EB energy E_EB_ to produce maximum output power P_EB_ for a fixed pulse duration t_EB_ and repetition frequency f_EB_ were as follows: E_EB_ = 5.5 MeV; I_EB_ = 130 mA; P_EB_ = 134 W (fEB = 50 Hz, tEB = 3.75 μs). The working parameters were adjusted to obtain a dose rate of ≈3.2 kGy/min at 60 cm from the electron exit window using for dosimetry a graphite calorimeter calibrated at Risø DTU National Laboratory for Sustainable Energy. The total accumulated doses for samples were 15 kGy. 

Regarding the FBGs, non-temperature treatment or any other treatment was performed before and after the exposure to radiation.

## 3. Results

After the irradiation, the samples were characterised using the same source and optical spectrum analyser (Figure 4a), as in Section 2, and the spectra were compared with measurements made prior to irradiation. We analysed the samples in terms of reflectivity, full width at half maximum (FWHM) bandwidth, coupling coefficient (*k*), refractive index modification ($∆n_{mod}$), and resonance wavelength with respect to the equation below,

(3) $$R_{max}=\tanh^{2}\left(kL\right)={\tan h}^{2}\left(\frac{\pi ∆n_{mod}\eta L}{m\cdot \lambda_{Bragg}}\right)\;$$

where L is the length of the grating, ***m*** is the order of the grating, and η is the mode overlap parameter between the cladding and core region.

Figure 4b,c show the spectra of the *PbP* and the *PlbPl* inscribed FBGs, respectively, before and after the exposure to γ-radiation. We analysed the grating profiles, and the results are summarised in Table 1 and Table 2, for the FBG inscribed using the *PbP* and the *PlbPl* method, respectively. For the gamma radiation, we observe a negative wavelength shift of −60 pm for the grating inscribed using the *PlbPl* method, whereas the *PbP* counterpart was only 6 pm. The *PbP*-FBG shows a decrease in the coupling coefficient (***k***) and the grating reflectivity. On the other hand, for the *PlbPl*-FBG, following the irradiation, both the reflectivity and ***k*** increased. 

**Figure 5:**
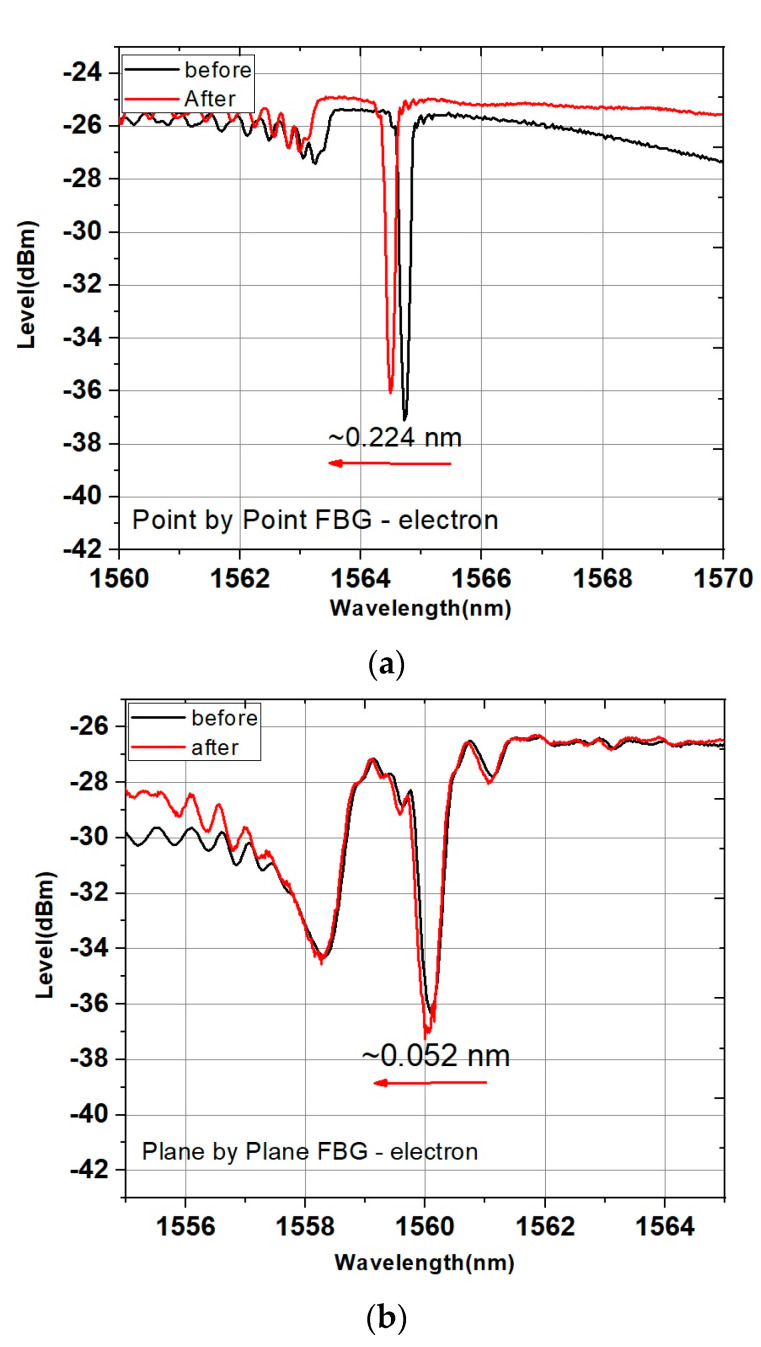
Transmission spectra of the FBG before and after the exposure to electron radiation, (**a**) *PbP-*inscribed FBG, (**b**) *PlbPl*-inscribed FBG.

Table 3 and Table 4 summarise the characteristics of the FBG samples exposed with electron radiation for *PbP* and *PlbPl* inscription methods, respectively, while the spectra are presented in Figure 5a,b, before and after their exposure. Again, we observe a decrease in reflectivity and ***k*** for the *PbP-*FBG and an increase with the *PlbPl*-FBG. However, it is noted that the *PbP*-FBG experiences a greater impact to the electron radiation with a −224 pm wavelength shift of the Bragg peak compared to 52 pm for the *PlbPl*. For the calculations of Table 1, Table 2, Table 3 and Table 4, we calculated η=0.82 and the relevant equations from reference [28].

### 3.1. Thermal Response

**Figure 6:**
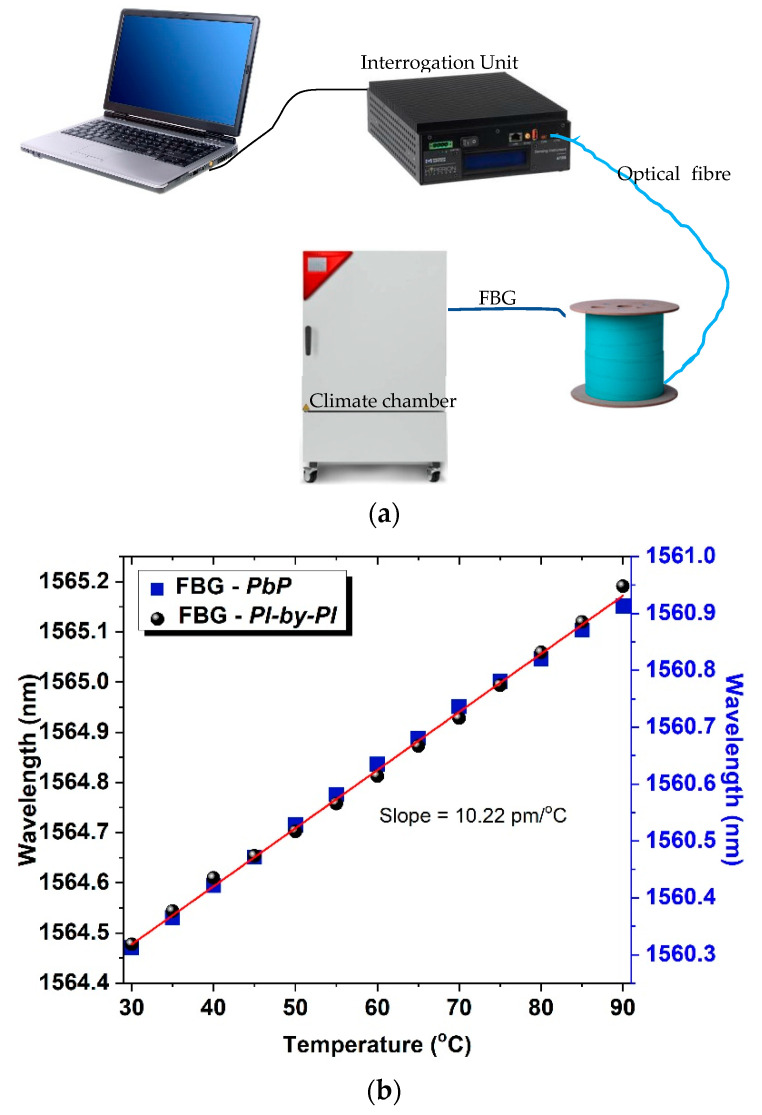
(**a**) Experimental schematic diagram used for the temperature characterisation of the FBGs, (**b**) Temperature response of femtosecond laser-inscribed FBGs using the *PlbPl* and the *PbP* method for temperature range of 30 to 90 °C.

After the exposure of the FBGs to electron and gamma radiation and their spectral characterisation, the samples were placed in a computer-controlled climate chamber for temperature measurements (Figure 6a). The temperature of the chamber was increased from 30 to 90 °C with steps of 5 °C, and the reflection spectrum of the gratings was measured using a Micron Optics Hyperion si155 with 1 pm resolution. 

As a reference sample, one FBG inscribed using the *PbP* and one using the *PlbPl* method was characterised under the specific temperature range, and their sensitivities are presented in Figure 6. The response for each of the two gratings for the same temperature range was exactly the same with a slope of 10.22 pm/°C. The thermal response of the FBGs was anticipated to be the similar, since the temperature response of the gratings reflects the properties of the material. 

**Figure 7:**
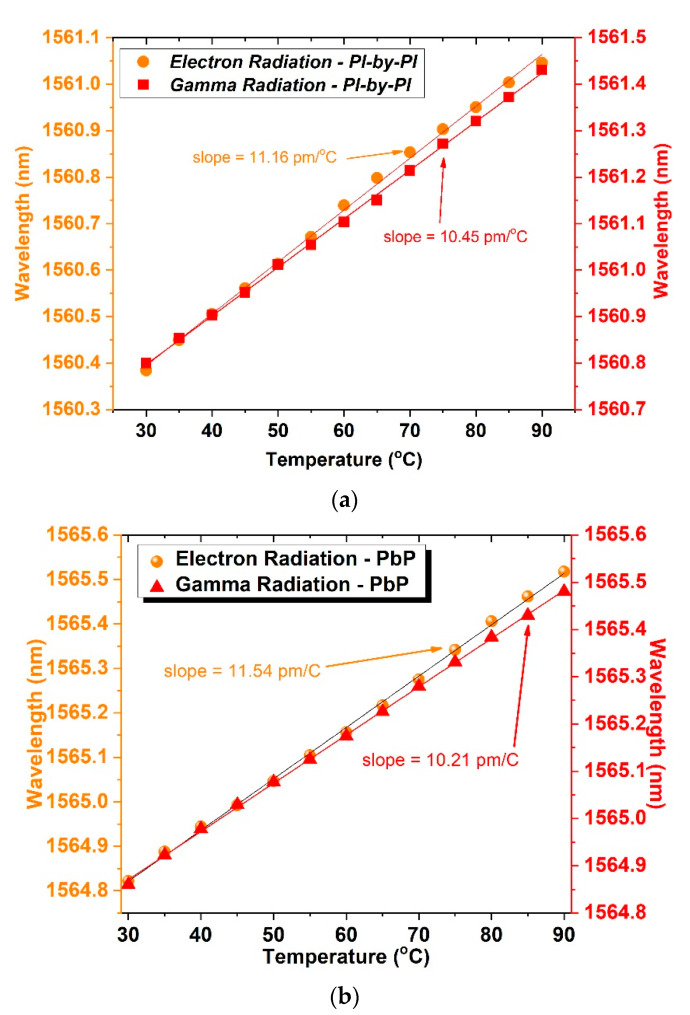
Temperature sensitivity of the FBGs after exposure to electron and gamma radiation, (**a**) *PlbPl*-inscribed FBG and (**b**) *PbP*-inscribed FBG.

In Figure 7, we present the temperature responses of the electron and gamma irradiated samples, for the grating inscribed with the *PlbPl* and *PbP* method, respectively. Comparing the results in two figures (Figure 7a,b), we observe a small increase in the value of the temperature coefficient of the *PlbPl*-FBG (10.45 pm/°C), whereas the *PbP* FBGs maintain their pre-irradiation values regarding the γ-radiation. On the other hand, the electron-radiated samples note a significant increase in the temperature coefficient. There is a 9.19% increase in value for the *PlbPl*-FBG and 12.91% for the *PbP*-FBG. 

### 3.2. FTIR Measurements

Fourier transform infrared (FTIR) spectroscopy was employed to analyse the molecular structure of the samples following irradiation. This technique evaluated the resultant spectra under changes in the vibrational modes of the molecules, where such information is used to verify and/or estimate the molecular structure by analysing and comparing the vibration in the frequency region of functional groups [29]. The source of the FTIR lies in the infrared (IR) range, and only the vibration modes that change the dipole moment of a molecule appear in the FTIR spectrum [30].

For the FTIR analysis, only the FBGs region of the fibre was used. The samples were crushed into powder and analysed using an attenuated total reflection (ATR) probe, which comprises a crystal plate and has an area of 1 mm^2^. In this region, the light source is focused, and the evanescent wave resulting from the total internal reflection on the sample is analysed. 

**Figure 8:**
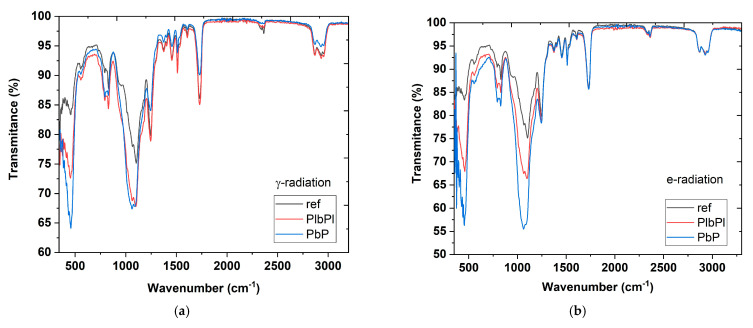

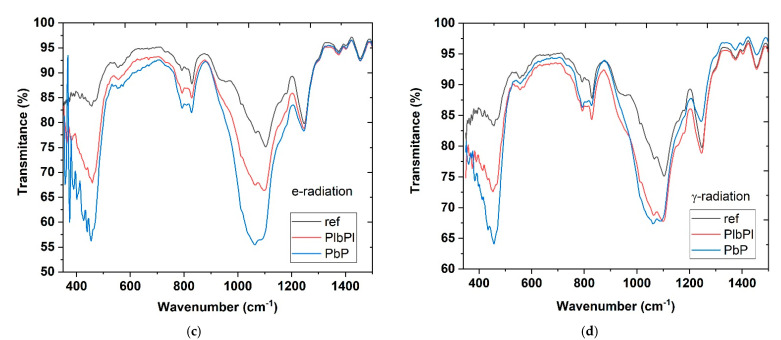
FTIR spectra of the irradiated (and reference) *PbP-* and the *PlbPl-*FBG samples when exposed to (**a**) γ-radiation and (**b**) e-radiation. The zoomed FTIR spectra of the exposed samples to (**c**) γ-radiation and (**d**) e-radiation.

To evaluate the influence of the irradiation in the samples’ molecular structure, the results of the FTIR for irradiated samples were compared with non-irradiated SMF28 optical fibre. In the region of 3200 to 340 cm^−1^, we have nine principal absorbance peaks, ≈1729.86, 1608.36, 1510, 1456, 1375, 1245, 1103, 829, and 457 cm^−1^. We can divide this spectrum in three regions, <500 cm^−1^, 500 to 1200 cm^−1^, and >1200 cm^−1^. The first region is related to the Germanium (Ge) bonds such as GeO_2_ and Ge-Si [31], the second region is related to silicon–oxygen bonds such as Si-OH and Si-O-Si for peaks higher than 1100 cm^−1^, while the last region is related to carbon bonds such as aliphatic CH groups and CO. In Figure 8a–d, we present the FTIR spectrum of the *PbP*-FBG and *PlbPl*-FBG samples irradiated with γ- and e-radiation, respectively, in comparison with a reference sample that was not exposed to any radiation. 

The major differences between the irradiated samples (when compared with the non-irradiated sample) are the peak intensities at the first two regions (i) <500 cm^−1^ and (ii) 500 to 1200 cm^−1^. Starting with the *PbP*- and *PlbPl*-FBG γ-radiated samples in Figure 8a,c, we observe similar changes at the spectra for the whole range except at the peaks located at 457 cm^−1^. Particularly, a small shift is observed to 453.2 cm^−1^ only for the *PlbPl*-FBG with peak intensity at 72.58%, while the peak transmittance for the *PbP*-FBG at 457 cm^−1^ dropped at 64.08% compared to 83.31% of the reference spectrum. There are also small shifts and changes on peaks at 829 cm^−1^ and 1100 cm^−1^, compared to the reference spectrum; however, these were very similar when the two FBG samples are compared. The changes in these peaks are possibly induced by the femtosecond laser radiation during the FBG inscription process. 

Similarly, Figure 8b,d present the FTIR results for both samples after exposure to electron radiation. In that case, we observe significant changes at two peaks, at ≈1103 cm^−1^ and at ≈457 cm^−1^. The transmittance peak at 1103 cm^−1^ with respect to the reference was shifted at 1058 cm^−1^ for the *PbP*-FBG sample and transmittance decreased to 55.66% compared to 75.12% of the reference sample. According the *PlbPl*-FBG sample, a frequency shift of −4 cm^−1^ was observed, which was significantly smaller when compared to the *PbP* sample, and the peak transmittance was measured at 66.42%. On the other hand, for the peak located at 457 cm^−1^ with respect to the reference spectrum, we observed a shift at 455 cm^−1^ for the *PbP*-FBG and at the 458 cm^−1^ for the *PlbPl*-FBG. The peak transmittance was decreased to 56.25% for the *PbP-*FBG and 67.9% for the *PlbPl*-FBG with respect to 83.31% of the reference spectrum. A comparison between the peak transmittance at 457 cm^−1^ between the γ- and e-radiation samples indicates that the electron radiation has a greater negative impact for both samples. Particularly, we note a peak decrease in the transmittance at 457 cm^−1^ for electron exposed samples of >12% and ≈5% respectively for the *PbP*- and *PlbPl*-FBG when compared with their FTIR spectra when exposed to γ-radiation. In addition, at 800 cm^−1^, when comparing the PbP- and PlbPl-FBG for the electron radiation, we see similar bandwidth and frequency peaks but difference transmittance.

## 4. Discussion

The comparison between the inscription methods, *PbP* and *PlbPl*, shows that the *PlbPl*-FBG leads to lower molecular changes after exposure to both types of radiation (electron and gamma), since the presented frequency shifts and intensities are closer to the reference sample. 

In Section 5, we note a significant change in the temperature coefficient for both samples, >9% for the *PlbPl-*FBG and >12% for the *PbP*-FBG, after the exposure to the electron radiation. On the other hand, for the samples exposed to gamma radiation, the temperature coefficient change was negligible. Given the FTIR results of the *PlbPl*- and the *PbP*-FBG samples for both electron and γ-radiation, we observe a common spectrum area that seems to be more susceptible to radiation exposure. This area is at 457 cm^−1^, which is related to the Ge-bonds. It is noted that the transmittance change at 457 cm^−1^ was higher for the *PbP*-FBG compared to the *PlbPl*-FBG. However, it seems that this area is not related with the temperature coefficient of the FBGs, since similar changes were observed for either sample exposed to electron or to gamma radiation. 

A comparison between *PbP* and *PlbPl* samples exposed to γ-radiation shows similar peak intensities and bandwidth at 1100 cm^−1^, which is related to the Si-bonds, and similar temperature coefficients for the two samples. Conversely, when comparing the FTIR data for the electron-radiated samples, the transmittance at 1100 cm^−1^ is different for the *PbP* and *PlbPl*-FBGs. Particularly, in respect to the γ-radiated samples, we had an increasing transmittance for the *PlbPl*-FBG of 2% and >10% for the *PbP*-FBG sample. As a result, it can be said that the significant change on the thermal coefficients of the FBGs is related with the molecular changes on the silicon–oxygen bonds at the 1100 cm^−1^ region and not with the Ge-bonds at 457 cm^−1^. In other words, it seems that both radiation types are breaking and re-establishing the bonds in different directions, particularly for Si-O. The rearrangement of the bonds can be verified by the frequency shift and bandwidth changes when compared to the reference sample. In addition, these variations on the molecular structures are related to changes in the physical properties and material responses, which includes the thermal parameters and temperature behavior. Moreover, changes in the 457 cm^−1^ region are possibly responsible for changes in the FBG profiles, since for all the gratings, we notice refractive index changes before and after the exposure. Particularly, we note changes on the FBG strength, bandwidth, and wavelength, which are all strongly related with the effective refractive index of the FBGs. 

Finally, it is well known that the temperature response of an FBG is the contribution of the thermal expansion and the thermo-optic effect. Following the previous discussion, it can be said that the changes in the thermal coefficient of an FBG after exposure to electron- and γ-radiation are principally related to changes in the thermal expansion of the fibre.

## 5. Conclusions

We performed electron and γ-irradiation directly on Bragg gratings-inscribed single mode fibres (SMF28) with ≈3% mol Germanium content, using the *PbP* and the *PlbPl* inscription method. The effect of the irradiation on the gratings according to the inscription method was studied for the first time to the authors’ knowledge. The total accumulated radiation dose for all fibre samples was 15 kG for both radiation types. The grating spectra were characterised before and after the irradiation, and the temperature response of all the samples were measured using a high-resolution spectrometer and a climate chamber for a temperature range between 30 and 90 °C. The results show that the e-radiation was more damaging for the fibre compared to the γ-radiation-exposed samples for both inscription methods. Differences were observed with the profile characteristics of the gratings, such as effective refractive index, bandwidth, and reflectivity, but also through the temperature response of the FBGs. We note a temperature sensitivity increase of 12.91% for the FBG inscribed using the *PbP* method after exposure to electron radiation compared to 9.19% for the *PlbPl* grating. The gratings inscribed using the *PlbPl* method were shown to have slightly better radiation hardness compared to the *PbP* gratings. Summarising all the results, we conclude that the thermal coefficient changes of the FBGs when exposed to ionised environments are more related to changes on the thermal expansion of the fibre (core and cladding) and material changes on the silicon–oxygen bonds, while modifications on germanium bonds are related with the optical characteristics of the FBGs.

## Figures and Tables

**Table 1:** Spectral characteristics of the *PbP*-FBG before and after exposure to γ-radiation.

	Before	After
**Reflectivity (dB)**	−6.43	−5.83
**Reflectivity (%)**	77.22	73.87
**FWHM (nm)**	0.144	0.144
***k***	150.33	128.33
**Δ*n_mod_***	3.5652 × 10^−4^	3.0434 × 10^−4^
**Δ*λ***	−6 pm	

**Table 2:** Spectral characteristics of the *PlbPl*-FBG before and after exposure to γ-radiation.

	Before	After
**Reflectivity (dB)**	−8.90	−9.33
**Reflectivity (%)**	87.11	88.83
**FWHM (nm)**	0.350	0.348
***k***	830.27	897.32
**Δ*n_mod_***	1.9640 × 10^−3^	2.1226 × 10^−3^
**Δ*λ***	−60 pm	

**Table 3:** Spectral characteristics of the *PbP*-FBG before and after exposure to electron radiation.

	Before	After
**Reflectivity (dB)**	−11.56	−11.10
**Reflectivity (%)**	93.80	92.23
**FWHM (nm)**	0.127 nm	0.119 nm
***k***	411.27	367.64
**Δ*n_mod_***	0.9752 × 10^−3^	0.8720 × 10^−3^
**Δ*λ***	−224 pm	

**Table 4:** Spectral characteristics of the *PlbPl*-FBG before and after exposure to electron radiation.

	Before	After
**Reflectivity (dB)**	−9.94	−10.77
**Reflectivity (%)**	89.86	91.61
**FWHM (nm)**	0.339	0.345
***k***	995.79	1792.49
**Δ*n_mod_***	2.3540 × 10^−3^	4.2400 × 10^−3^
**Δ*λ***	−52 pm	
